# FAD Mutations in Amyloid Precursor Protein Do Not Directly Perturb Intracellular Calcium Homeostasis

**DOI:** 10.1371/journal.pone.0011992

**Published:** 2010-08-05

**Authors:** Emily Stieren, Walter P. Werchan, Amina El Ayadi, Fuzhen Li, Darren Boehning

**Affiliations:** 1 Department of Neuroscience and Cell Biology, University of Texas Medical Branch, Galveston, Texas, United States of America; 2 Mitchell Center for Neurodegenerative Diseases, University of Texas Medical Branch, Galveston, Texas, United States of America; University of North Dakota, United States of America

## Abstract

Disturbances in intracellular calcium homeostasis are likely prominent and causative factors leading to neuronal cell death in Alzheimer's disease (AD). Familial AD (FAD) is early-onset and exhibits autosomal dominant inheritance. FAD-linked mutations have been found in the genes encoding the presenilins and amyloid precursor protein (APP). Several studies have shown that mutated presenilin proteins can directly affect calcium release from intracellular stores independently of Aβ production. Although less well established, there is also evidence that APP may directly modulate intracellular calcium homeostasis. Here, we directly examined whether overexpression of FAD-linked APP mutants alters intracellular calcium dynamics. In contrast to previous studies, we found that overexpression of mutant APP has no effects on basal cytosolic calcium, ER calcium store size or agonist-induced calcium release and subsequent entry. Thus, we conclude that mutated APP associated with FAD has no direct effect on intracellular calcium homeostasis independently of Aβ production.

## Introduction

Alzheimer's disease (AD) is a progressive neurological disorder characterized by deterioration of cognitive abilities. AD is the most common cause of dementia in the western world, affecting one in ten individuals over 65 and nearly 50% of all persons over the age of 85 [1], [2]. According to the predominant amyloid cascade hypothesis, AD pathogenesis is associated with a series of molecular events which leads to the extracellular deposition and aggregation of specific proteolytic fragments of APP. These aggregated protein fragments constitute the core of extracellular senile amyloid plaques, a pathologic hallmark of AD.

In normal physiology, APP is cleaved by a series of enzymes, called secretases, generating proteolytic fragments of various lengths. The principal cleavage event is by α-secretase, which generates a large soluble ectodomain (APP_s_) that is secreted into the extracellular space and a C-terminal fragment (C83) that remains in the membrane [3]. In an alternate processing pathway, holo-APP can be cleaved by β-secretase, resulting in the production of a secreted ectodomain and the membrane-associated C99 fragment [3]. Subsequent cleavage of C99 by γ-secretase produces the neurotoxic Aβ peptide and an intracellular domain (AICD) that is released from the membrane into the cytosol. There are two major forms of the Aβ peptide that differ in length by 2 residues, Aβ_40_ and Aβ_42_. The Aβ_42_ peptide is more prone to aggregation and is considered to be more cytotoxic than the shorter Aβ species [4].

Most cases of AD are sporadic and late-onset, but rare forms of familial AD (FAD) are early-onset and exhibit autosomal-dominant inheritance. The majority of FAD cases are linked to mutations in the presenilin (PS) genes 1 and 2 [5]. Presenilins constitute the catalytic core of γ-secretase, and PS mutations lead to relative overproduction of Aβ_42_ [6], [7], [8], [9]. FAD-linked mutations have also been found in APP, and depending on the mutation result in increased β-secretase processing, increased Aβ_42_/Aβ_40_ ratio, increased propensity of Aβ to form fibrils, or decreased proteolytic clearance of Aβ peptides [10], [11], [12], [13], [14], [15], [16], [17], [18], [19], [20].

While it is clear that APP-derived fragments are involved in a proximal step in the pathogenesis of AD, the exact mechanism of neuronal loss in not known. Also, clinical symptoms do not correlate well with amyloid plaque load, suggesting that a certain level of neuronal dysfunction precedes gross architectural changes in AD brain [21], [22]. Calcium dyshomeostasis has been implicated as a major contributor to neuronal cell death in AD [23]. Calcium dynamics regulate Aβ production, and Aβ peptides/fibrils directly affect multiple aspects of calcium homeostasis [24]. There is strong evidence that mutated presenilin proteins can directly modulate calcium release from intracellular stores independently of Aβ production [25], [26], [27], and may also form calcium permeable channels in the endoplasmic reticulum [28]. Similarly, several studies have suggested that APP may directly modulate calcium homeostasis independently of Aβ production [29], [30], [31]. However, a systematic study of the effects of FAD-associated APP mutants on intracellular calcium homeostasis has not been reported.

Here, we show that overexpression of FAD-linked APP mutants has no effect on basal cytosolic calcium concentration, ER calcium store size, or agonist-induced calcium release and subsequent entry. These results indicate that mutant APP likely does not contribute mechanistically to alterations in calcium homeostasis in AD independently of Aβ production.

## Results

### Expression of Different FAD-linked APP Mutants in PC12 Cells

**Figure 1:**
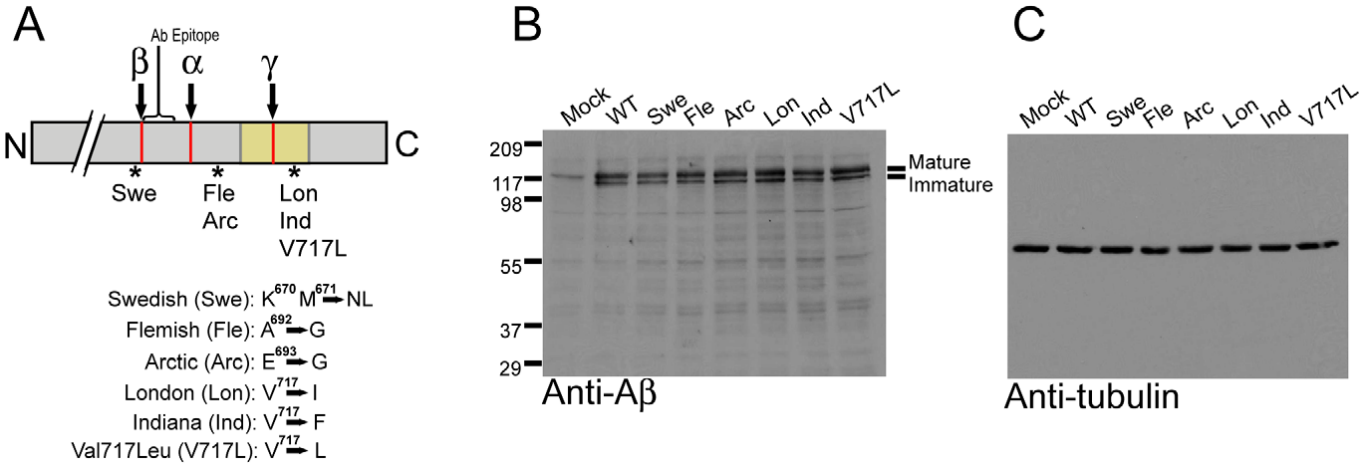
Expression of wild-type APP and different FAD-linked APP mutants in PC12 cells. (A) Schematic diagram depicting the C-terminal portion of APP with secretase cleavage sites indicated in red and locations of FAD-linked mutations marked with asterisks. The transmembrane region is shaded in yellow. The epitope for the anti-Aβ 1–10 antibody (Millipore catalogue # 07-592) is indicated. Specific amino acid substitutions for each mutation are shown with residue numbering corresponding to APP_770_. (B) Overexpression of APP constructs in PC12 cells. Mock cells were co-transfected with YFP and empty vector. (C) Immunoblot for α/β-tubulin to demonstrate equal loading.

For our studies, we focused on six different well-characterized FAD-linked APP mutants that affect β-secretase cleavage, fibrillization, and γ-secretase cleavage (**Figure 1A**). The Swedish double mutant makes APP a more favored substrate for β-secretase shunting full-length APP down the amyloidogenic processing pathway [10], [11]. The London, Indiana, and V717L mutations favor production of Aβ_42_ by the γ-secretase [12], [13], [14], [15], [16]. The Flemish and Arctic APP mutations increase the propensity for fibrillization and decrease proteolytic clearance of Aβ peptides [17], [18], [19], [20].

Immunoblot analysis of PC12 cells overexpressing wild-type and mutant APP revealed two bands with approximate molecular weight of 110–120 kDa corresponding to immature and mature forms of the holoprotein (**Figure 1B**). As APP is trafficked through the secretory pathway, a series of glycosylation events occur leading to a mature, or fully glycosylated, protein with slower mobility on SDS-PAGE. An immunoblot for α/β-tubulin is shown as a loading control (**Figure 1C**).

### ER Calcium Release, Receptor-operated Calcium Entry, and ER Calcium Store Size Are Not Affected by FAD-linked Mutant APP

**Figure 2:**
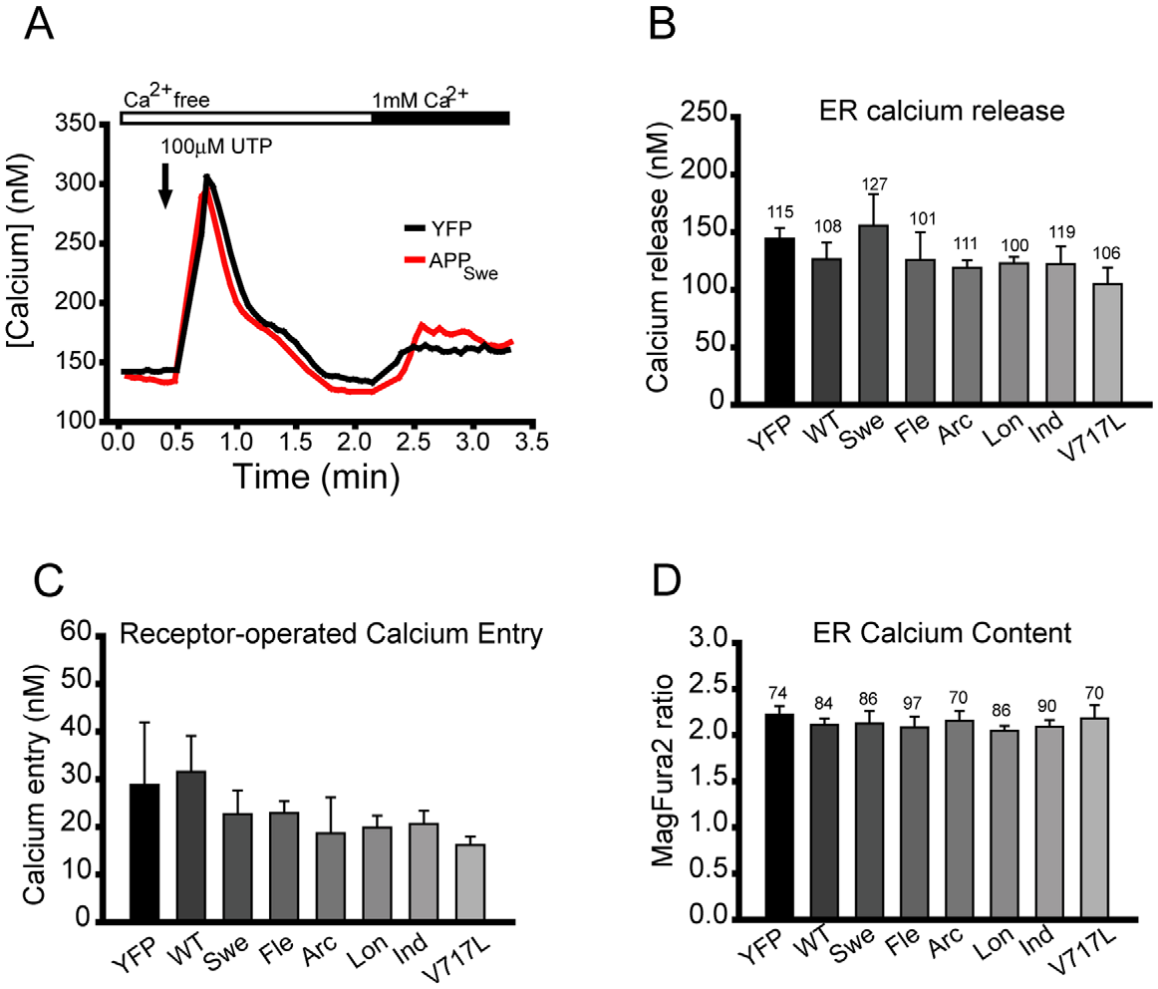
ER calcium release, store-operated entry, and store size are not affected by overexpression of APP mutants. (A) Representative single cell traces of cytosolic [Ca^2+^] in control cells (black) and cells expressing APP with the Swedish mutation (red). (B) Peak calcium release in response to 100 µM UTP in calcium-free medium. The total number of single cells imaged for each condition is indicated above the error bar. All cells were co-transfected with either empty vector or APP and yellow fluorescent protein (YFP) at a 4∶1 ratio. WT, wild-type; Swe, Swedish; Fle, Flemish; Arc, Arctic; Lon, London; Ind, Indiana. (C) Store-operated calcium entry following addition of calcium-replete medium in control and APP-transfected cells for each of the APP constructs tested. These results are from continuous imaging of the same coverslips used for the calcium release experiments shown in (A). (D) ER store size in control and APP-transfected cells for each of the APP constructs tested. For all experiments, error bars represent standard error of the mean. There was no statistical significance between control and APP expressing cells (p>0.05 for all conditions).

Calcium release from ER-resident inositol 1,4,5-trisphosphate receptors (IP_3_Rs) has been proposed to be a critical mediator of calcium dyshomeostasis in AD [25], [26], [27]. To determine whether mutant APP expression affects IP_3_R-mediated calcium release, we measured the response of PC12 cells to the purinergic agonist UTP, which selectively activates phospholipase C-coupled P2Y receptors [32]. PC12 cells were co-transfected with yellow fluorescent protein (YFP) and one of the following: empty vector control, wild-type APP, or one of six FAD-linked APP mutants. To selectively examine IP_3_R activity and exclude the contribution of calcium entry from the plasma membrane, UTP stimulation was done in calcium free media. As indicated in the representative fura-2 calcium traces in **Figure 2A**, addition of UTP induces a robust calcium release from the ER into the cytosol in calcium-free media. Peak calcium release in response to UTP did not differ between control cells expressing empty vector, cells expressing wild-type APP, and cells expressing APP mutants (**Figure 2B**).

Depletion of ER calcium stores by agonist stimulation triggers store/receptor-operated calcium entry (SOC/ROC) through channels in the plasma membrane [33]. Entry through these channels has been proposed to be compromised in fibroblasts expressing mutant presenilins [34]. To determine the effect of FAD-linked APP mutants on receptor-operated calcium entry, we waited until cytosolic calcium levels reached baseline following UTP stimulation in calcium-free medium and replaced the bath solution with calcium-replete medium. As shown in **Figure 2C**, receptor-operated calcium entry did not differ significantly between control cells expressing YFP alone, cells expressing wild-type APP, and cells expressing any of the six APP mutants tested.

Next, we wanted to determine the effect of FAD-linked mutant APP on ER calcium store size. To directly measure ER calcium content, we utilized ER compartmentalized mag-fura-2 [35], [36], [37]. As shown in **Figure 2D**, ER calcium store size did not differ between control cells, wild-type APP-expressing cells, and any of the six APP mutants tested.

### Agonist-Induced Calcium Release Is Not Affected by FAD-linked Mutant APP

**Figure 3:**
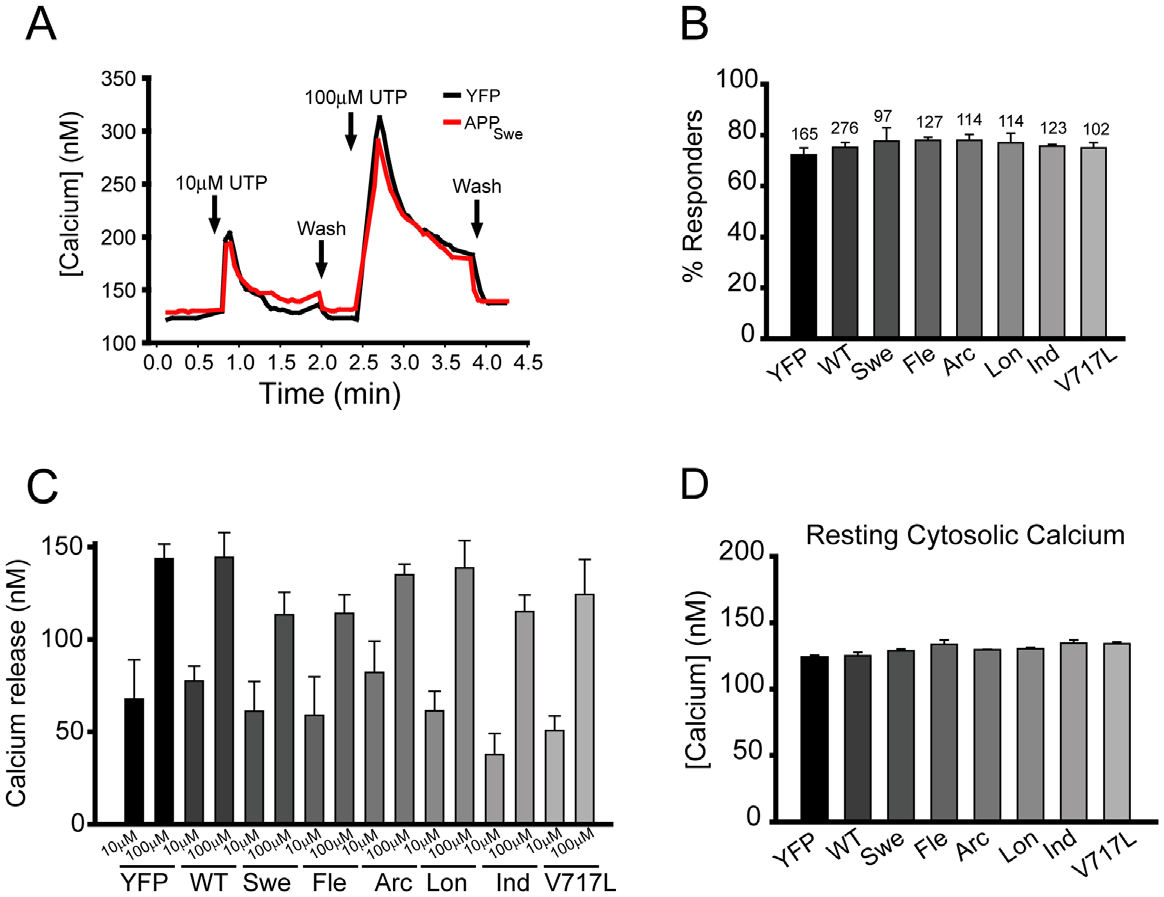
Agonist-induced calcium entry is not affected by overexpression of FAD-linked APP mutants. (A) Representative single cell traces of cytosolic [Ca^2+^] in cells expressing YFP alone (control) or APP with the Swedish mutation (red) upon addition of 10 µM and 100 µM UTP. (B) Percentage of cells that responded to 10 µM UTP for each condition. (C) Quantification of peak calcium release induced by UTP. (D) Resting (basal) cytosolic calcium concentration in control and APP-transfected cells for each of the APP constructs tested. Error bars represent standard error of the mean. There was no statistical significance between control and APP expressing cells (p>0.05 for all conditions).

Mutant presenilins directly modulate calcium release from ER stores [25], [26], [27]. To address whether mutant APP has similar effects, we monitored the response of PC12 cells to two different doses of UTP. As shown in the representative traces in **Figure 3A**, addition of subsaturating (10 µM) and saturating (100 µM) doses of UTP resulted in transient increases in cytosolic calcium. There was no difference in the percent of cells that responded to the subsaturating dose (**Figure 3B**), indicating that there were no significant differences in sensitivity to agonist-induced calcium release. There were also no differences observed in peak calcium release induced by either dose between control, wild-type APP, and the six mutant APP-expressing cells (**Figure 3C**). By analyzing baseline calcium levels in each condition, we were also able to determine that resting cytosolic calcium was not altered in cells expressing wild-type APP or FAD-linked mutant APP (**Figure 3D**).

## Discussion

The results of these studies indicate that FAD-linked APP mutants do not directly affect calcium homeostasis independently of Aβ production, at least in this model system. Several studies have shown that overexpression of wild-type APP or FAD-linked mutant APP leads to an increase in basal cytosolic calcium concentration and an increase in ER store size. Rojas and colleagues demonstrated that neurons derived from mice possessing three copies of the APP gene have increased resting cytosolic calcium concentration and altered responses to glutamatergic and nicotinic agonists and that these effects could be partially restored by APP knockdown [29]. Cortical neurons from the triple-transgenic (3xTg-AD) mouse model of AD were also shown to have elevated resting cytosolic calcium [30]. Overexpression of the Swedish APP mutant alone was sufficient to cause the same effect. In these studies, cells were taken from young animals prior to plaque development, suggesting that the changes observed represent proximal events in the disease model. In cells from the 3xTg-AD mice, calcium entry as well as release from intracellular stores both contributed to elevated cytosolic calcium levels [30].

It has been shown that agonist-induced calcium release from intracellular stores is disrupted in fibroblasts from APP null mice [31]. This effect is related to a decrease in ER store size and can be rescued by APP constructs containing the APP intracellular domain (AICD), suggesting that this peptide fragment is involved in regulating intracellular calcium stores [31]. Subsequent studies showed that the loss of AICD was associated with decreased levels of ATP, perhaps impairing ER calcium uptake *via* the sarco-endoplasmic reticulum calcium ATPase [38]. Other studies have shown that wild-type APP is not directly involved in modulating calcium stores but does mediate the increase in store size that is seen with certain PS mutants and PS deficiency [39]. The effect of PS knockdown on store size was exacerbated in cells expressing the FAD-linked APP mutant V717I, and this effect correlated with increased levels of C99 in these cells [39].

Here, we demonstrated that FAD-linked APP mutations do not directly alter calcium signaling when overexpressed in PC12 cells. These results suggest that a different mechanism must exist for the perturbations in intracellular calcium that are observed in forms of FAD that are linked to mutations in APP. Most likely, these effects are mediated directly by Aβ and not the APP holoprotein. Furthermore, our results promote the hypothesis that the disruption in calcium signaling seen with PS mutants is independent of the role for PS in APP cleavage, and may instead reflect a general role for PS in the maintenance of intracellular calcium homeostasis.

## Methods

### Cell Lines

PC12 rat pheochromocytoma cells were purchased from ATCC. They were cultured in Dulbecco's Modified Eagle's Medium supplemented with 10% fetal bovine serum, 5% horse serum, 100 U/ml penicillin, 100 µg/ml streptomycin.

### Generation of APP Mutants

Point mutations in human APP_695_ (kindly provided by Dr. Hui Zheng, Ph.D., Baylor College of Medicine) were accomplished with the QuickChange Site-directed Mutagenesis Kit (Stratagene, La Jolla, CA) according to manufacturer's instructions. Forward primers were as follows:

Swedish 5′-GGAGATCTCTGAAGTGAACCTGGATGCAGAATTCC-3′;

Flemish 5′-CAAAAATTGGTGTTCTTTGGAGAAGATGTGGG-3′;

Arctic 5′-GGTGTTCTTTGCAGGAGATGTGGGTTCAAAC-3′;

London 5′-CATAGCGACAGTGATCATCATCACCTTGGTGATGC-3′;

Indiana 5′-CATAGCGACAGTGATCTTCATCACCTTGGTGATGC-3′;

V717L 5′-CATAGCGACAGTGATCCTCATCACCTTGGTGATGC-3′.

The reverse primers were the exact reverse complement.

### Calcium Imaging

Calcium measurements were performed as previously described [40]. PC12 cells were cultured on 25 mm coverslips overnight, and were co-transfected with APP and yellow fluorescent protein (YFP) at a 4∶1 ratio. Under these conditions, we have found that all YFP-positive cells express both proteins [41]. For all experiments, transfection efficiency as determined by fluorescence microscopy was greater than 50%. Similar results were obtained by imaging APP-GFP fusion proteins (data not shown). For measurement of cytosolic calcium, cells were loaded with fura-2 as described elsewhere [40] for 30 minutes at 25°C. For ER calcium measurements, cells were loaded with 5 µM mag-fura-2 for 20 minutes at 37°C followed by a 60-minute incubation at 37°C in dye-free extracellular medium (107 mM NaCl, 7.2 mM KCl, 1.2 mM MgCl_2_, 1 mM CaCl_2_,11.5 mM glucose, 0.1% bovine serum albumin, and 20 mM HEPES 7.2). Coverslips were transferred to the microscope and the plasma membrane was subsequently permeabilized by a brief exposure to 0.01% saponin to release cytoplasmic dye before exchanging the solution with intracellular solution (135 mM KCl, 3 mM MgATP, 2 mM MgCl, 0.4 mM CaCl_2_, 1 mM EGTA, and 20 mM HEPES 7.1) as described [35]. All fields were imaged randomly, and all YFP-positive cells in a given field were imaged and quantified in the data analysis. The number of cells analyzed is indicated above each data point. Fura-2/mag-fura-2 and YFP images were acquired every 3 seconds during acquisition on a Nikon TE2000 inverted microscope using a Nikon 60X oil immersion SuperFluor objective with a 1.3 numerical aperture. All imaging was performed at 25°C. Images were captured with a Roper Scientific CoolSNAP HQ monochrome camera. Rapid filter changes for ratiometric imaging were computer controlled *via* a Ludl MAC6000 rapid filter wheel and changer and MetaFluor data acquisition and analysis software. Raw data was acquired with MetaFluor, analyzed in Excel, and graphed in Sigma Plot.

### Statistical Analysis

All experiments were performed a minimum of three times and presented as the mean ± standard error. Single cell traces from each coverslip were pooled and averaged for each data point. Total number of single cell traces is indicated over each bar. Statistical comparisons from the pooled data were performed between groups using the student's *t* test. Statistical significance was considered at p values <0.05.

## References

[1] Hebert LE, Scherr PA, Bienias JL, Bennett DA, Evans DA (2003). Alzheimer disease in the US population: prevalence estimates using the 2000 census.. Arch Neurol.

[2] Evans DA, Funkenstein HH, Albert MS, Scherr PA, Cook NR (1989). Prevalence of Alzheimer's disease in a community population of older persons. Higher than previously reported.. Jama.

[3] De Strooper B, Annaert W (2000). Proteolytic processing and cell biological functions of the amyloid precursor protein.. J Cell Sci.

[4] Finder VH, Glockshuber R (2007). Amyloid-beta aggregation.. Neurodegener Dis.

[5] Selkoe DJ (2005). Defining molecular targets to prevent Alzheimer disease.. Arch Neurol.

[6] Wolfe MS, Xia W, Ostaszewski BL, Diehl TS, Kimberly WT (1999). Two transmembrane aspartates in presenilin-1 required for presenilin endoproteolysis and gamma-secretase activity.. Nature.

[7] Borchelt DR, Thinakaran G, Eckman CB, Lee MK, Davenport F (1996). Familial Alzheimer's disease-linked presenilin 1 variants elevate Abeta1-42/1-40 ratio in vitro and in vivo.. Neuron.

[8] Citron M, Westaway D, Xia W, Carlson G, Diehl T (1997). Mutant presenilins of Alzheimer's disease increase production of 42-residue amyloid beta-protein in both transfected cells and transgenic mice.. Nat Med.

[9] Scheuner D, Eckman C, Jensen M, Song X, Citron M (1996). Secreted amyloid beta-protein similar to that in the senile plaques of Alzheimer's disease is increased in vivo by the presenilin 1 and 2 and APP mutations linked to familial Alzheimer's disease.. Nat Med.

[10] Mullan M, Crawford F, Axelman K, Houlden H, Lilius L (1992). A pathogenic mutation for probable Alzheimer's disease in the APP gene at the N-terminus of beta-amyloid.. Nat Genet.

[11] Haass C, Lemere CA, Capell A, Citron M, Seubert P (1995). The Swedish mutation causes early-onset Alzheimer's disease by beta-secretase cleavage within the secretory pathway.. Nat Med.

[12] Murrell JR, Hake AM, Quaid KA, Farlow MR, Ghetti B (2000). Early-onset Alzheimer disease caused by a new mutation (V717L) in the amyloid precursor protein gene.. Arch Neurol.

[13] Suzuki N, Cheung TT, Cai XD, Odaka A, Otvos L (1994). An increased percentage of long amyloid beta protein secreted by familial amyloid beta protein precursor (beta APP717) mutants.. Science.

[14] Murrell J, Farlow M, Ghetti B, Benson MD (1991). A mutation in the amyloid precursor protein associated with hereditary Alzheimer's disease.. Science.

[15] Tamaoka A, Odaka A, Ishibashi Y, Usami M, Sahara N (1994). APP717 missense mutation affects the ratio of amyloid beta protein species (A beta 1-42/43 and a beta 1-40) in familial Alzheimer's disease brain.. J Biol Chem.

[16] Goate A, Chartier-Harlin MC, Mullan M, Brown J, Crawford F (1991). Segregation of a missense mutation in the amyloid precursor protein gene with familial Alzheimer's disease.. Nature.

[17] De Jonghe C, Zehr C, Yager D, Prada CM, Younkin S (1998). Flemish and Dutch mutations in amyloid beta precursor protein have different effects on amyloid beta secretion.. Neurobiol Dis.

[18] Nilsberth C, Westlind-Danielsson A, Eckman CB, Condron MM, Axelman K (2001). The ‘Arctic’ APP mutation (E693G) causes Alzheimer's disease by enhanced Abeta protofibril formation.. Nat Neurosci.

[19] Hendriks L, van Duijn CM, Cras P, Cruts M, Van Hul W (1992). Presenile dementia and cerebral haemorrhage linked to a mutation at codon 692 of the beta-amyloid precursor protein gene.. Nat Genet.

[20] Kamino K, Orr HT, Payami H, Wijsman EM, Alonso ME (1992). Linkage and mutational analysis of familial Alzheimer disease kindreds for the APP gene region.. Am J Hum Genet.

[21] Braak H, Braak E (1991). Neuropathological stageing of Alzheimer-related changes.. Acta Neuropathol.

[22] Duyckaerts C, Hauw JJ (1997). Diagnosis and staging of Alzheimer disease.. Neurobiol Aging.

[23] LaFerla FM (2002). Calcium dyshomeostasis and intracellular signalling in Alzheimer's disease.. Nat Rev Neurosci.

[24] Green KN, LaFerla FM (2008). Linking calcium to Abeta and Alzheimer's disease.. Neuron.

[25] Leissring MA, Parker I, LaFerla FM (1999). Presenilin-2 mutations modulate amplitude and kinetics of inositol 1, 4,5-trisphosphate-mediated calcium signals.. J Biol Chem.

[26] Leissring MA, Paul BA, Parker I, Cotman CW, LaFerla FM (1999). Alzheimer's presenilin-1 mutation potentiates inositol 1,4,5-trisphosphate-mediated calcium signaling in Xenopus oocytes.. J Neurochem.

[27] Cheung KH, Shineman D, Muller M, Cardenas C, Mei L (2008). Mechanism of Ca2+ disruption in Alzheimer's disease by presenilin regulation of InsP3 receptor channel gating.. Neuron.

[28] Nelson O, Tu H, Lei T, Bentahir M, de Strooper B (2007). Familial Alzheimer disease-linked mutations specifically disrupt Ca2+ leak function of presenilin 1.. J Clin Invest.

[29] Rojas G, Cardenas AM, Fernandez-Olivares P, Shimahara T, Segura-Aguilar J (2008). Effect of the knockdown of amyloid precursor protein on intracellular calcium increases in a neuronal cell line derived from the cerebral cortex of a trisomy 16 mouse.. Exp Neurol.

[30] Lopez JR, Lyckman A, Oddo S, Laferla FM, Querfurth HW (2008). Increased intraneuronal resting [Ca2+] in adult Alzheimer's disease mice.. J Neurochem.

[31] Leissring MA, Murphy MP, Mead TR, Akbari Y, Sugarman MC (2002). A physiologic signaling role for the gamma -secretase-derived intracellular fragment of APP.. Proc Natl Acad Sci U S A.

[32] von Kugelgen I (2006). Pharmacological profiles of cloned mammalian P2Y-receptor subtypes.. Pharmacol Ther.

[33] Putney JW (2007). Recent breakthroughs in the molecular mechanism of capacitative calcium entry (with thoughts on how we got here).. Cell Calcium.

[34] Leissring MA, Akbari Y, Fanger CM, Cahalan MD, Mattson MP (2000). Capacitative calcium entry deficits and elevated luminal calcium content in mutant presenilin-1 knockin mice.. J Cell Biol.

[35] Solovyova N, Fernyhough P, Glazner G, Verkhratsky A (2002). Xestospongin C empties the ER calcium store but does not inhibit InsP3-induced Ca2+ release in cultured dorsal root ganglia neurones.. Cell Calcium.

[36] Hofer AM, Machen TE (1993). Technique for in situ measurement of calcium in intracellular inositol 1,4,5-trisphosphate-sensitive stores using the fluorescent indicator mag-fura-2.. Proc Natl Acad Sci U S A.

[37] Mogami H, Tepikin AV, Petersen OH (1998). Termination of cytosolic Ca2+ signals: Ca2+ reuptake into intracellular stores is regulated by the free Ca2+ concentration in the store lumen.. EMBO J.

[38] Hamid R, Kilger E, Willem M, Vassallo N, Kostka M (2007). Amyloid precursor protein intracellular domain modulates cellular calcium homeostasis and ATP content.. J Neurochem.

[39] Herms J, Schneider I, Dewachter I, Caluwaerts N, Kretzschmar H (2003). Capacitive calcium entry is directly attenuated by mutant presenilin-1, independent of the expression of the amyloid precursor protein.. J Biol Chem.

[40] Boehning D, van Rossum DB, Patterson RL, Snyder SH (2005). A peptide inhibitor of cytochrome c/inositol 1,4,5-trisphosphate receptor binding blocks intrinsic and extrinsic cell death pathways.. Proc Natl Acad Sci U S A.

[41] Wozniak AL, Wang X, Stieren ES, Scarbrough SG, Elferink CJ (2006). Requirement of biphasic calcium release from the endoplasmic reticulum for Fas-mediated apoptosis.. J Cell Biol.

